# Enrichment of a set of microRNAs during the cotton fiber development

**DOI:** 10.1186/1471-2164-10-457

**Published:** 2009-09-29

**Authors:** Pieter Bas Kwak, Qin Qin Wang, Xu Sheng Chen, Cheng Xiang Qiu, Zhi Min Yang

**Affiliations:** 1 Department of Biochemistry and Molecular Biology, College of Life Science, Nanjing Agricultural University, Nanjing, PR China; 2 Laboratory of Cotton Breeding, Industrial Crop Institute, Jiangsu Academy of Agricultural Sciences, Nanjing, PR China

## Abstract

**Background:**

Cotton (*Gossypium hirsutum*) is one of the most important economic crops and provides excellent fibers for textile manufacture. In addition to its industrial and agricultural importance, the fiber cell (plant trichome) also is a biological model system for exploring gene expression and regulation. Small RNAs regulate many aspects of plant growth and development. However, whether small RNAs are involved in regulation of fiber cell development is unknown.

**Results:**

We adopted a deep sequencing approach developed by Solexa (Illumina Inc.) to investigate global expression and complexity of small RNAs during cotton fiber initiation and development. We constructed two small RNA libraries prepared from wild type (WT) and fuzz/lintless (*fl *Mutant in the WT background) cotton ovules, respectively. Each library was sequenced individually and generated more than 6-7 million short sequences, resulting in a total of over 13 million sequence reads. At least 22 conserved candidate miRNA families including 111 members were identified. Seven families make up the vast majority of expressed miRNAs in developing cotton ovules. In total 120 unique target genes were predicted for most of conserved miRNAs. In addition, we identified 2 cell-type-specific novel miRNA candidates in cotton ovules. Our study has demonstrated significant differences in expression abundance of miRNAs between the wild-type and mutant, and suggests that these differentially expressed miRNAs potentially regulate transcripts distinctly involved in cotton fiber development.

**Conclusion:**

The present study is the first to deep sequence the small RNA population of *G. hirsutum *ovules where cotton fibers initiate and develop. Millions of unique miRNA sequences ranging from 18~28 nt in length were detected. Our results support the importance of miRNAs in regulating the development of different cell types and indicate that identification of a comprehensive set of miRNAs in cotton fiber cells would facilitate our understanding of the regulatory mechanisms for fiber cell initiation and elongation.

## Background

MicroRNAs (miRNAs) are a class of short (~21 nt), endogenous non-coding small RNAs that have base pair sequences complementary with specific target genes to repress their translation or induce their degradation. While in animals regulation of gene expression by miRNAs is achieved by sequence-specific targeting of the 3' untranslated region of mRNAs, the plant miRNAs generally interact with their targets through perfect or near-perfect complementarity to direct mRNA degradation [1,2]. A growing number of new miRNAs in plants have been identified in recent years. To date, more than 1000 genes encoding miRNAs have been annotated in *Arabidopsis*, rice and other plant species [3]. Moreover, several other classes of small RNAs (also known as small interacting RNAs, siRNAs), distinguished by their origin and biological function, have been identified. These include heterochromatic siRNAs, *trans*-acting siRNAs (ta-siRNAs), natural antisense siRNAs (nat-siRNAs), Piwi-interacting RNAs [4], and a recently identified class of small RNAs associated with gene promoters (PASRs) and 3' termini (TASRs) [5].

Identification of comprehensive sets of miRNAs and other small RNAs in different plant species is a critical step to facilitate our understanding of regulatory mechanisms or networks for target genes and cell development. The higher plants contain diverse cell types. Each of them has its own initiating program, structure and biological function. Cellular differentiation is accompanied by changes in transcription, translation and many other physiological processes [6]. Cotton fibers are single-celled epidermal trichomes and provide an outstanding model for investigation of cellular and developmental events which also occur in Arabidopsis leaf trichomes [7]. The development of a fiber cell is a complex morphological and molecular process, which is characterized by cell cycle status, transcriptional control and multiple cytoskeletal functions comprising an integrated hierarchy of regulation. Recently, a number of genes controlling early fiber initiation and late development have been identified, and some of them have been functionally characterized. *GhMYB109*, a putative ortholog of *AtMYBGL1*, is specifically expressed in fiber cell initials and elongating cells [8]. Moreover, ectopic expression of *GaMYB2 *induces a single trichome in epidermis of Arabidopsis seeds [9]. Two cotton genes containing WD40 domains complement the Arabidopsis *ttg1 *mutant [10]. Comparative studies on *Arabidopsis *leaf trichomes and multiple gene expression have provided great insights into the cotton ovular fiber development [6,11-13]. Using microarray technology, hundreds of transcripts were analyzed and exhibited distinct expression patterns during the early stage of fiber cell development [6,11-16]. Using a computational approach, we initially identified 37 potential miRNAs from cotton (*G. hirsutum*); further, 96 potential targets were detected for these potential miRNAs [17]. More recently, several other labs also performed *in silico *identification of dozens of conserved miRNAs from the same species [18-20]. Amongst the putative targets these studies reported were transcription factors (*e.g*. MYB), auxin responsive proteins and other genes related to fiber development [17,18]. To explore the role of small RNAs in cotton fibers, Abdurakhmonov and co-workers recently analyzed small RNA sequences from 0-10 days post anthesis (DPA) developing cotton ovules and obtained hundreds of small RNAs [21]. Although 583 unique sequence signatures of small RNAs were achieved, only two conserved miRNAs were detected. It is most likely that the traditional method does not sequence deeply enough to sample the full complexity of small RNAs in ovules. Recently developed high-throughput sequencing technologies provide a powerful approach to identify and quantify sRNAs/miRNAs [22]. Small RNAs are best discovered and measured by deep sequencing methods that have high sensitivity and specificity [23-25]. Additionally, it is feasible to explore or annotate miRNAs in organisms whose genome sequences have not been completed. Here, we adopted a deep sequencing method developed by Solexa (Illumina Inc.) to identify small RNAs from cotton ovules and analyze abundance and complexity of small RNAs. We constructed two small RNA libraries prepared from wild type (WT) and fuzz/lintless (Mutant in the same background) cotton ovules, respectively. Samples were collected from 0-10 DPA developing cotton ovules, which cover major morphological changes as well as several underlying developmental processes including fiber initiation and elongation [11]. Each library was sequenced individually and generated more than 6-7 million short sequences, with a total of over 13 million sequence reads. We obtained more than 100 conserved miRNAs representing 36 families from the cotton ovules. Many of them were originally identified in this study. In addition, two non-conserved novel miRNA candidates were identified.

## Results

### Analysis of sequences

**Figure 1:**
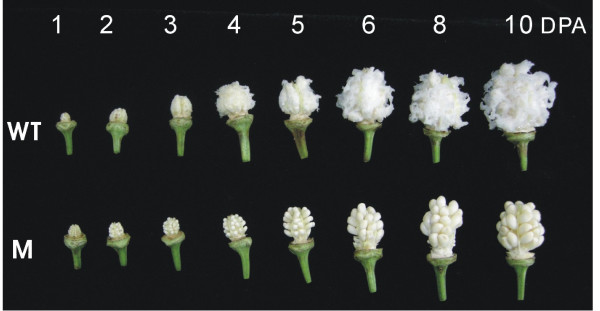
**Developing wildtype and mutant ovules**. Excised ovaries exhibiting developing ovules from *Gossypium hirsutum *Xu-142 wildtype (WT) and *fuzzless-lintless *mutant (M)(in Xu-142 background), harvested at different days post anthesis (DPA). The young cotton trichomes from the wildtype have been slightly and gently plucked open with a pair of tweezers to exemplify and visualize the presence and length of the rapidly elongating trichomes that are virtually absent in the mutant.

**Figure 2:**
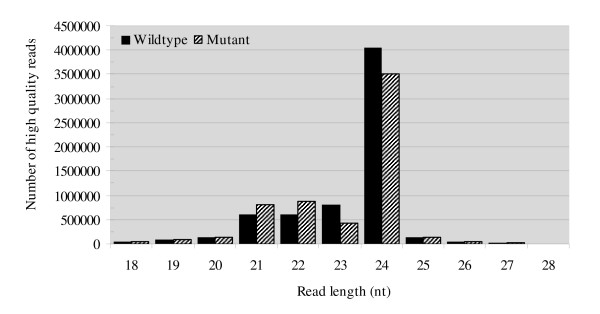
**Abundance of small RNA sequences with different size derived from wild-type and mutant libraries**. All of the reads are of high quality, ranging from 18-28 nt in length.

Previous studies have demonstrated that cotton fiber development is a complex process that involves a large number of gene expression and regulation [6,7,26]. To understand whether small RNA is involved in the process, we employed a upland cotton cultivar Xuzhou 142 (wild type) and a *fuzzless-lintless *mutant in Xuzhou 142 background, both of which were genetically identified [27] and are phenotypically similar except for the feature of the mutant seeds bearing few or no fibers (Figure 1). Microarray analyses of transcripts demonstrated that a number of genes in the wild-type and *fl *mutant ovules were differentially expressed between 0-10 DPA [11]. Based on the fact, we reasoned that deep sequencing ovular small RNA would provide a full view of small RNA components and differential expression profiles of small RNAs between the wild-type and mutant. Thus, two small RNA libraries were constructed from the wild-type and mutant ovules. Deep sequencing the libraries generated 7,055,692 and 6,517,694 sequence reads from the wild-type and mutant ovules, respectively. After the removal of low quality reads and corrupted adapter sequences (reads < 18 nt and reads > 28 nt), 6,584,945 reads (4,124,219 unique sequences) remained for the wild-type and 6,069,470 reads (3,391,661 unique sequences) for the mutant. The majority of small RNAs was 21-24 nt for both libraries (> 90%), with 24 nt small RNA being the most abundant (Figure 2, Additional file 1), which is within the typical size range for Dicer-derived products and in agreement with most of the previous results [28-30].

The dataset was used to query the non-coding RNAs sequences deposited at NCBI GenBank  and the Rfam database [3] in order to separate the small RNAs that match to non-coding sequences such as rRNA, tRNA, snRNA and snoRNA. These accounted for 332,455 reads (46,542 unique sequences) in wild-type and 292,158 reads (45,612 unique sequences) in mutant. Since the cotton genome has not been completely sequenced, we used Short Oligonucleotide Analysis Package (SOAP, )[31] to annotate small RNA sequences that map to TIGR Cotton Transcript Assemblies (TIGR) . With only 70,667 TAs available, less than 10% of the small RNAs from each library were mapped.

### Identification of conserved candidate miRNAs

**Figure 3:**
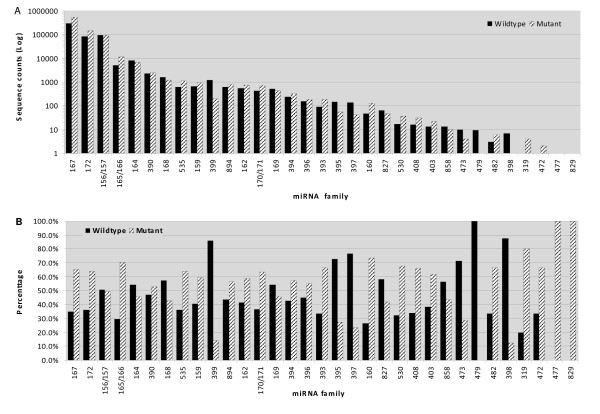
**The relative abundance and differential expression levels of identified candidate miRNA families**. A: The number of sequence counts reflects the relative abundance of a miRNA family. B: miRNA differential expression levels represented as a percentage of the total family sequence count (WT+M). miRNA families have been sorted on abundance to facilitate overview. The differential expression of highly abundant families are more likely to resemble actuality.

**Figure 4:**
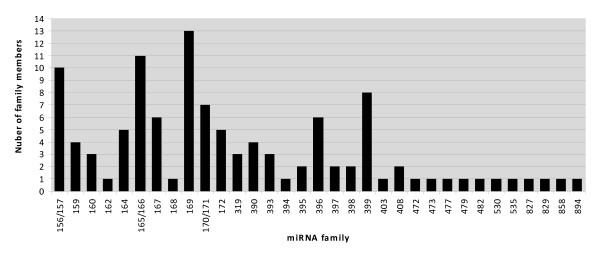
**Number of detected family members per miRNA family**. Candidate miRNA families were taken together and grouped by their miRBase  numerical identifiers.

Aligning small RNA sequences to known miRNAs resulted in 496,654 and 816,359 matches for wild-type and mutant, respectively. At least 22 conserved potential miRNA families including 111 individual candidate miRNAs were identified, with miRNA families 167, 172 and 156/157 being the most abundant (Table 1, Figure 3, Additional file 2). MiR167 dominated in both libraries and accounted for 59.1% (WT) and 66.8% (M) of all conserved miRNA reads. Several miRNA families such as miR159, miR162 and miR894 had moderate abundance of expression. In contrast, some miRNA families showed very low abundance of expression in ovules, with several read counts only. The varied abundance of the miRNA families suggests that miRNA genes would be differentially transcribed during the early fiber cell development. Diversity of cotton fiber miRNAs also can be found in the aspect of the amount of members they contain. The largest miRNA family size identified was miR169 that consisted of 13 members and miR165/166, miR156/157 and miR399 possessed 11, 10 and 8 members, respectively, whereas 16 miRNA families such as miR162, miR170 and miR394 had only one member detected in the cotton ovules (Figure 4).

**Table 1:** Identified known (or conserved) candidate miRNA families in *Gossypium hirsutum *wildtype (WT) and fuzz/lintless mutant (M) ovules.

**miRNA Family**	**Sequence Reads**		**Ratio**	
			
	**WT**	**M**	**(WT/M)**	
**156/157**	96705	94568	1.0	**
159*	642	949	0.7	**
**160**	46	129	0.4	**
**162**	542	762	0.7	**
**164**	8183	6863	1.2	**
**165/166**	4951	11909	0.4	**
**167**	293960	545537	0.5	**
**168**	1633	1208	1.4	**
**169**	523	441	1.2	**
170*	1	1	1.0	
**171**	433	750	0.6	**
**172**	83392	147672	0.6	**
319*	1	4	0.3	
**390**	2258	2518	0.9	**
**393**	93	184	0.5	**
**394**	232	313	0.7	**
**395**	150	56	2.7	**
**396**	155	191	0.8	**
**397**	137	42	3.3	**
**398**	7	1	7.0	
**399**	1222	202	6.0	**
**403**	13	21	0.6	**
**408**	16	31	0.5	**
**472**	1	2	0.5	
473*	10	4	2.5	
477*	0	1	0.0	
479*	9	0	x	**
**482**	3	6	0.5	
530*	17	36	0.5	**
535*	634	1115	0.6	**
**827**	65	47	1.4	
**829**	0	1	0.0	
858*	13	10	1.3	
894*	607	785	0.8	**

*Newly identified miRNAs from *Gossypium hirsutum*. **p < 0.01.

Amongst the conserved miRNAs families, ten (consisting of 15 individual candidate miRNAs) have not been identified before in cotton by any of the earlier, mostly *in silico*, studies [17-19,21,32]. These miRNAs can be considered as new but species-conserved miRNAs in *G. hirsutum*. These included miR159a/b/c/f, miR170, miR319a/c/e, miR473a, miR477, miR479, miR530, miR535a, miR858 and miR894. (Table 1, Additional file 2). We also compared miRNAs from this study to those computationally predicted and found there were 22 overlaps of miRNA families (Additional file 3), indicating that most of the previously predicted miRNAs could be recovered by the deep-sequencing method.

The number of reads reflects enrichment of miRNAs. Most of the miRNA read frequencies exhibit significant differences between the two libraries. Expression of miR165/166, miR159, miR160, miR162, miR167, miR171, miR172, miR390, miR393, miR394, miR396, miR403, miR408, miR503, miR535 and miR894 were significantly up-regulated in mutant compared to the wild-type, whereas miR156/157, miR164, miR168, miR169, miR395, miR397 and miR399 showed down-regulation in mutant relative to the wild-type. The abundantly presented families like miR165/166, miR160 and miR167 were expressed very highly in the mutant. MiR399 was detected 6-fold higher in wild-type than in mutant. Several other miRNA families such as miR393, miR408 and miR530 also showed higher levels of expression in mutant than in wild-type. To a lesser degree, miR395 and miR397 showed comparatively low expression levels in mutant. Interestingly, the highly enriched miR156/157 family was found at similar levels in both libraries.

### Analysis of novel miRNA candidates

**Figure 5:**
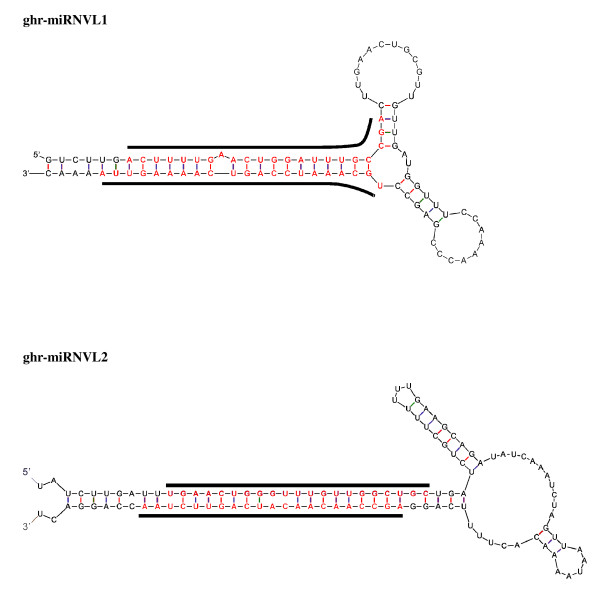
**Mature and precursor sequences and the predicted stem-loop structures of newly cloned miRNAs from *G. hirsutum***. The mature miRNAs are in red and underlined. The actual size of the precursors may be slightly shorter or longer than represented.

Since the completely sequenced genome of cotton is unavailable, unique small RNA sequences were mapped to cotton TIGR Plant Transcript Assemblies sequences to identify potentially novel miRNAs. Also, because of the unknown background of cotton small RNA population, it is rather challenging to confidentially identify non-conserved miRNAs. Secondary structures were predicted and analyzed for stable stem-loop hairpins. Following BLASTn search and hairpins structure prediction, two putative *G. hirsutum *unique miRNAs were detected (Figure 5), both of which meet the new criteria of miRNA annotation [25]. We found that the two miRNAs (named ghr-miRNVL1 and ghr-miRNVL2) have structures that feature both miRNA and miRNA* (Table 2, Figure 5). For ghr-miRNVL1, 1074 reads were detected at the 5' and 9 reads at the 3'. Ghr-miRNVL2 had 313 5' reads and 3 3' reads. Ghr-miRNVL1 and ghr-miRNVL2 were found in both wild-type and mutant libraries, suggesting that the 2 miRNA candidates are *G. hirsutum*-specific.

**Table 2:** Read frequency and mature sequences of *Gossypium hirsutum *novel miRNA candidates.

**ID**	**Precursor**	**Library**	**5p Reads**	**3p Reads**	**5p Sequence (5' -- 3')** **3p Sequence (5' -- 3')**
miRNVL1	AI054573	WT	324	5	**ACTTTTGAACTGGATTTGCCGA(22)**
		M	750	4	**TGCAAATCCAGTCAAAAGTTA(21)**
miRNVL2	DW497660	WT	79	3	**TGAACTGGGTTTGTTGGCTGC(21)**
		M	234	0	**AGCCAACAACATCAGTTCTAA(21)**

The 5p and 3p sequences originate from the 5' and 3' arm of the folded precursors, respectively. WT: Xuzhou 142 (wild-type); M: *fuzzless-lintless *mutant in Xuzhou 142 background.

### Prediction of miRNA targets

Targets were predicted for all identified miRNA families. In total 120 unique target genes were predicted for 21 of the conserved miRNA families (Additional file 4). Only genes with known or putative functions were presented. Some miRNA families have multiple target sites (*e.g*. miR399g), suggesting that these miRNAs are functionally divergent. Additionally, a single gene may potentially be targeted by several miRNAs (*e.g*. miR171a/b/f). On the basis of the biological functions described by UniProt , these target genes can be grouped into 10 categories. The majority of targets fall into the category of transcription regulation, indicating these genes encode transcript factors (Table 3). Several other groups contain genes regulating transport, oxidative reduction, signal transduction pathway, and enzymes involved in metabolisms, respectively. Unfortunately, none of the target genes have been predicted for the two novel candidate miRNAs. This is most possibly attributed to the incomplete cotton genome.

**Table 3:** Predicted target functions for the identified cotton conserved candidate miRNAs.

**Gene function**	**Number of targets**
Regulation of transcription	54
Oxidation reduction	13
Transporter	11
Metabolism	6
Transcription	6
Proteolysis	5
Signal transduction	5
Lipid metabolism	4
Stress response	4
Other	11
Total	119

Targets were predicted for the identified conserved miRNA (see Additional file 4) and grouped by the biological function of the proteins they encode for, as described by UniProt . Transcripts coding for proteins involved in the regulation of transcription were prevalent.

## Discussion

To date, more than one thousand plant miRNA genes have been annotated and some of them have been well characterized [3]. However, the number of plant miRNAs appears not to be saturated and many other functional miRNAs in plant species remain to be investigated. Compared to annotated miRNAs from Arabidopsis and rice, very few miRNAs from cotton plants have been identified. Recently, several studies performed *in silico *identification of miRNAs from *G. hirsutum *[17-19,32]. Approximate 18 highly conserved miRNA families were detected and several less conserved miRNAs (or families) were found. When compared to the miRNAs predicted previously, most of them could be recovered by deep sequencing, and only a small portion of them (*e.g*. miR391 and miR400) were not [17-19,32]. These missing miRNAs might be attributed to the fact that the sequences (*e.g*. EST or GSS) used for prediction were derived from tissues such as leaves or roots rather than ovules. Also, it was likely that false positive predictions were included. On the other hand, several miRNAs (or families) such as miR159, miR172, miR319, miR473, miR477, miR479, miR530, miR535, miR858 and miR894 were not successfully predicted, suggesting that these miRNAs may be tissue-specific in cotton ovules. MiR172 and miR390 have been recently cloned from cotton (*G. hirsutum*) ovule using a traditional cloning approach [32], both of which were also detected in this study.

The present study is the first to deep sequence the small RNA population of *G. hirsutum *ovules where cotton fiber cells initiate and develop. Millions of unique siRNA sequences ranging from 18~28 nt in length were detected. Analysis of the evolutionary conservation of these miRNAs revealed 111 individual conserved miRNAs belonging to 22 families. Together with the several *G. hirsutum *miRNAs existing in miRBase (Release 12.0, Sept, 2008, www.sanger.ac.uk/Software/Rfam/ftp.shtml), this study will bring the number of miRNAs in *G. hirsutum *up to 120.

The vast majority of conserved miRNAs from cotton ovules is not surprising. Most of the miRNAs identified in this study are conserved in Arabidopsis and only a few are conserved in other plant species. The phenomenon can be explained by the fact that cotton fibers (seed trichomes) and epidermal hairs (leaf trichomes) are phenotypically similar; both types of trichomes use a common mechanism, *e.g*. that closely associated with the transcription factors for regulating trichome initiation and development [9,16]. Notably, some highly conserved miRNA families such as miR156/157, miR167 and miR172 were sequenced more than ten thousands or even one hundred thousands times in a single library. These highly conserved miRNAs may represent a relationship between evolutionary conservation and expression abundance [24]. On the opposite, some miRNA families that are less conserved or even species-specific have very low abundance. From an evolutionary view, these miRNAs play a role in establishing and maintaining phenotypic diversity between different groups of organisms and are involved in regulation of the lineage-specific pathways and functions [24,33]. In addition to the conserved miRNAs, two putative miRNAs identified in this study do not have orthologs in Arabidopsis and other species (Table 2). Since the non-conserved miRNAs usually express at a low level and in specific cell types (ovules), it is suggested that these cotton-specific miRNAs may have expanded after the divergence of the monocot and dicot plant lineages, supporting the presumption that a diverse set of miRNAs are evolving rapidly and independently with each species [34].

Our comparative analysis of small RNA abundance between the wild-type and *fl *mutant indicated that the mutant contains an altered small RNA population, with a smaller proportion of total RNA reads. However, in *fl *mutants many small RNA families with 21-22 nt were enriched (Figure 2). Differential miRNA abundance was also found between the wild-type and mutant. A surprising observation was that the majority of miRNA families in *fl *mutant had significantly higher abundance than in wild-type (Figure 3B). This result suggests that the mutant has a changed regulation of *MIRNA *expression during the fiber development. Further identification of the regulatory process and metabolic pathway in mutants will provide insights into the impaired miRNA biogenesis and abnormal trichome cell differentiation.

It is of interest that a large number of miRNAs from ovules potentially target transcription factors, which was consistent with our previous predictions [17]. In *Arabidopsis*, miR159 mediates cleavage of *MYB101 *and *MYB33 *transcripts, the two transcription factors that function as positive regulators of abscisic acid (ABA) response [35]. Similarly, miR319 is complementary to a highly conserved motif in the coding region of the GAMYB-related clade of *MYB *[36,37]. In this study, several members of putative MYB families were predicted to have binding sites for miR169, miR396, miR399 and miR858, suggesting the potential regulation of fiber development. MiR858 is of particular interest because it targets the *GhMYB10 *mRNA. Ectopic expression of *GhMYB10 *in transgenic tobacco plants causes abnormal cell shapes of leaf trichomes [38].

Amongst the predicted targets of miR167 was Auxin Response Factors (ARF). These proteins are bound to the auxin response elements and regulate auxin-mediated transcriptional activation/repression. *In vitro*-cultured cotton ovules, exogenous auxin is required to promote fiber cell development [26]. Our data have demonstrated that in the mutant, miR167 was expressed more highly. In rice culture cells, miR167 was shown to cleave ARF8 mRNA. The abundance of miR167 was controlled by the level of auxin in growth medium. When cells were grown in auxin-free medium, miR167 level decreased [12]. Similarly, a putative ARF8 transcript was predicted to be targeted by miR167 in cotton ovules. This suggests that auxin levels are possibly higher in the mutant ovules. We predicted miR160 to target an ARF10-like mRNA transcript, which expressed at higher levels in the mutant library, just like miR167. MiR393 targets putative Transport Inhibitor Response 1 (TIR1) transcripts in cotton. TIR1 is an auxin receptor involved in a mechanism leading to the Aux/IAA degradation [39]. Inhibition of TIR1 by miR393 would down-regulate auxin signaling. MiR393 showed an expression level nearly 2 fold higher in mutants than in wild-type. Interestingly, most of the miRNAs involved in the auxin pathway were found to be up-regulated in the mutant.

In Arabidopsis, several miRNAs like miR399 and miR395 are induced by the nutrient deficiency [40,41]. Under normal growth conditions, these miRNAs do not express. However, both miR399 and miR395 were moderately sequenced in this study, particularly in the wild-type ovules (Table 1). This can be attributed to the advantage that deep sequencing can detect miRNAs at a very low level. Under phosphorus starvation, miR399 targets a ubiquitin-conjugating E2 enzyme, which in turn regulates Pi acquisition [41,42]. In this study, deep sequencing identified 8 miR399 members and 7 unique genes were predicted as potential targets of miR399. More interestingly, all of these predicted targets appear not to be correlated with phosphate metabolism. This result provides a new clue to the multiple roles of miR399 that may play in diverse cell types or species. MiR399f/g were predicted to have complementarity to a putative MYB family transcription factor. Besides, miR399g targets four cotton vacuolar ATP synthase subunit B transcripts. In cotton fiber, elongation is driven by turgor pressure generated by vacuolar H^+^-ATPase activity on tonoplasts [43]. The process occurs synchronously with the increase in the rate of cell elongation, indicating that vacuolar H^+^-ATPase may play a crucial role in cotton fiber development [44].

Expression of miR398 was much lower in mutant than in wild-type. Previously, miR398 in Arabidopsis was identified to target gene coding Cu/Zn superoxide dismutase [45]. Similar target was predicted for the miR398 from cotton ovules. Interestingly, specific cotton Cu/Zn superoxide dismutase have been recently detected in the secondary cell walls of developing cotton fibers and are suggested to be involved in cell wall growth [46]. Whether miR398 regulates superoxide dismutase and cell wall growth would be an interesting topic to be investigated.

## Conclusion

Using deep sequencing method many of conserved miRNAs from cotton ovules were identified. Our results indicated that there are differential expression profiles of miRNAs from the wild-type and mutant ovules, which can be expected to regulate transcripts distinctly involved in cotton fiber development. Further identification of these differentially expressed miRNAs from ovules would allow better understanding of the regulatory mechanisms for fiber cell development.

## Methods

### Plant materials

Upland cotton plants (*Gossypium hirsutum *L.) cv. Xuzhou 142 (wild type; WT) and *fuzzless-lintless *mutant (M) in Xuzhou 142 background were field grown at the Jiangsu Agricultural Academy of Sciences under regular field conditions during spring/summer 2008. Flowers were tagged and developing ovules were harvested and directly dissected from 0 to 10 DPA ovaries in early mornings. The excised ovules were frozen in liquid nitrogen and stored at -80°C for analysis. Wild type and mutant ovules of 1, 2, 3, 4, 5, 6, 8, 10 DPA were selected for total RNA isolation.

### Total RNA isolation

Ovular total RNA was extracted using the pBiozol Total RNA Extraction Reagent (BioFlux) according to the manufacturer's instructions, supplemented with two extra chloroform washes before nucleic acid precipitation. A 1% agarose gel, stained by ethidium bromide, was run to preliminarily indicate the integrity of the RNA. All RNA samples were quantified and examined for protein contamination (A260 nm/A280 nm ratios) and reagent contamination (A260 nm/A230 nm ratios) by a Nanodrop ND 1000 spectrophotometer. In addition, the RIN (RNA integrity number) determined by the Agilent Technologies 2100 Bioanalyzer was greater than 8 for all samples.

### Small RNA library construction and sequencing

Total RNA from wild type and mutant was prepared for small RNA Sequencing-by-Synthesis according to the prescribed procedure and standards of the Illumina Sample Preparation Protocol. The samples were quantified and equalized so that equivalent amounts of RNA from mutant and wild-type were analyzed. In brief, total RNA was purified by electrophoretic separation on a 15% TBE-urea denaturing PAGE gel and small RNA regions corresponding to the 18-30 nucleotide bands in the marker lane were excised and recovered. The 18-30 nt small RNAs were 5' and 3' RNA adapter-ligated by T4 RNA ligase and at each step length validated and purified by urea PAGE gel electrophoretic separation. The adapter-ligated small RNA was subsequently transcribed into cDNA by SuperScript II Reverse Transcriptase (Invitrogen) and PCR amplified, using primers that anneal to the ends of the adapters. The amplified cDNA constructs, too, were purified and recovered. The final quality of the library was ensured by validation of the size, purity and concentration of the cDNA library on an Agilent Technologies 2100 Bioanalyzer. The two constructed cDNA libraries subsequently underwent Solexa/Illumina's proprietary flow-cell cluster generation and bridge amplification. After which the 1G sequencer, during automated cycles of extension, recorded fluorophore excitation and determined the sequence of bases for each cluster.

### Analysis of sequencing data

Raw sequence reads were produced by the Illumina 1G Genome Analyzer at BGI-Shenzhen, China and processed into clean full length reads by the BGI small RNA pipeline. During this procedure all low quality reads, including 3' adapter reads and 5' adapter contaminants were removed. The remaining high quality sequences were trimmed of their adapter sequences and sequences larger than 30 nt and smaller than 18 nt were discarded. All high quality sequences, even those with only a single unique read, were considered as significant and further analyzed. Unique small RNA sequences were mapped to cotton TIGR reference sequences by SOAP [31]. Small RNAs derived from rRNAs, tRNAs, snRNAs and snoRNAs deposited at the Rfam and NCBI GenBank databases  were identified by NCBI blast. In order to determine conserved miRNAs, unique sequences were aligned with known miRNAs from miRBase (Release 12.0, Sept, 2008)[3] with a maximum of two mismatches, where gaps count as mismatches. Potentially novel miRNAs were identified by folding the flanking genome sequence of unique small RNAs using MIREAP , followed by the prediction of the secondary structure by mFold 3.1 [47]. The essential criteria [25] were used for selecting the miRNA candidates, *e.g*. sequences of miRNA precursors can fold into a hairpin secondary structure that contains the ~21 nt mature miRNA sequence from one arm and miRNA* derived from the opposite arm, both of which form a duplex with two nucleotide, 3' overhangs. For prediction of miRNA targets, the procedure and criteria were followed as described previously [17,48]. More strictly, at most three mismatches between miRNA sequences and potential mRNA targets were allowed in this study. The biological function of the predicted targets was retrieved from the Universal Protein Resource .

### Statistical analysis

We used the chi-squared test to determine the statistical significance of the differences between the two libraries and applied the Yates correction for one degree of freedom. Our null hypothesis is based on that between the wild-type (expected frequency) and the mutant (observed frequency) there is no significant difference. In order to do so we normalized the total mutant sequence reads to the total high quality reads of the wild-type library. This approach allowed us to determine whether the deviations (the difference between observed and expected) were the result of chance, or whether they were due to other factors. In the case of a calculated probability p < 0.01 we reject our null hypothesis and conclude that a factor other than chance is operating for the deviation to be so great.

## Authors' contributions

PBK carried out ovule small RNA isolation, participated in the computational analyses and drafted manuscript. QQW carried out the molecular genetic studies. XSC carried out field cotton plant cultivation and ovule collection. CXQ participated in the sequence alignment. ZMY conceived of the study, participated in its design, and drafted and amended the manuscript. All authors read and approved the final manuscript.

## Supplementary Material

Additional file 1 **Additional Figure S1**. Percentage of small RNA sequences with different size derived from wild-type and mutant libraries. All of the reads are of high quality, ranging from 18-28 nt in length. Click here for file

Additional file 2 **Additional Table S2: Identified known candidate miRNAs from *Gossypium hirsutum *wild-type (WT) and fuzz/lintless mutant (M) ovules**. "Sequence" stands for the most occurring read that is homologue to the known miRNA. Click here for file

Additional file 3 **Additional Table S3**. Overlaps of miRNAs between the previously published computational miRNA predictions and those found in this study. Click here for file

Additional file 4 **Additional Table S4: Predicted targets for identified candidate miRNAs. miRNAs targeting the same gene and site are grouped together**. The first listed miRNA has the least mismatches. "Mm" stands for the total amount of mismatches between the first mentioned miRNA and the predicted target. "Alignment" visually represents miRNA/mRNA complementary base-pairs and mismatches for the first listed miRNA, with vertical bars and spaces as Watson-Crick base-pairs and mismatches, respectively (G:U wobbles count as mismatches). Click here for file
