# Supplementary material for: Enrichment of a set of microRNAs during the cotton fiber development
Source: BMC Genomics. 2009 Sep 29;10:457. doi: 10.1186/1471-2164-10-457 (PMC2760587; doi:10.1186/1471-2164-10-457)
Supplement: Additional file 1 — Additional Figure S1. Percentage of small RNA sequences with different size derived from wild-type and mutant libraries. All of the reads are of high quality, ranging from 18-28 nt in length. [file 1471-2164-10-457-S1.DOC]

**Additional Figure 1:**

**Percentage of small RNA sequences with different size derived from wild-type and mutant libraries.** All of the reads are of high quality, ranging from 18-28 nt in length.
