# Supplementary material for: Enrichment of a set of microRNAs during the cotton fiber development
Source: BMC Genomics. 2009 Sep 29;10:457. doi: 10.1186/1471-2164-10-457 (PMC2760587; doi:10.1186/1471-2164-10-457)
Supplement: Additional file 2 — Additional Table S2: Identified known candidate miRNAs from Gossypium hirsutum wild-type (WT) and fuzz/lintless mutant (M) ovules. "Sequence" stands for the most occurring read that is homologue to the known miRNA. [file 1471-2164-10-457-S2.DOC]

**Additional Table 2:**

**Identified conserved candidate miRNAs from *Gossypium hirsutum* wildtype (WT) and fuzz/lintless mutant (M) ovules.**

| **Family** | **miRNA**** | **Sequence** | **Sequence Reads** | |
| --- | --- | --- | --- | --- |
|  |  |  | **WT** | **M** |
| **156** | 156a | UGACAGAAGAGAGUGAGCAC | 1313 | 405 |
|  | 156b | CUGACAGAAGAUAGAGAGCAC (3) | 169 | 300 |
|  | 156c | UUGACACAAGAGAGAGAGCAC(3) | 18 | 57 |
|  | 156d | UUGACAGAAGAGAGGGAGCAC(2) | 13 | 18 |
|  | 156g | CGACAGAAGAGAGAGAGCAC(1) | 3 | 2 |
|  | 156h | UUGACAGAAGAGAGAGAGCAU(3) | 19 | 27 |
|  | 156k | UGACAGAAGAGAGAGAGCAC(1) | 460 | 734 |
|  | 156l | CGACAGAAGAGAGUGAGCACA(1) | 1 | 1 |
| **157** | 157a | UUGACAGAAGAUAGAGAGCAC(0) | 91156 | 89275 |
|  | 157d | UGACAGAAGAUAGAGAGCAC(0) | 3553 | 3749 |
| **159*** | 159a | UUUGGAUUGAAGGGAGCUCUA(0) | 634 | 938 |
|  | 159b | UUUGGAUUGAAGGGAGCUCUU(0) | 8 | 8 |
|  | 159c | UUUGGAUUGAAGGGAGCUCC(0) | 0 | 1 |
|  | 159f | CUUGGAUUGAAGGGAGCUCUA(0)r | 0 | 2 |
| **160** | 160a | UGCCUGGCUCCCUGUAUGCCA(0) | 39 | 108 |
|  | 160f | UGCCUGGCUCCCUGAAUGCCA(0) | 7 | 20 |
|  | 160h | UGCCUGGCUCCCUGCAUGCCA(0)p | 0 | 1 |
| **162** | 162a | UCGAUAAACCUCUGCAUCCAG | 542 | 762 |
| **164** | 164a | UGGAGAAGCAGGGCACGUGCA(0) | 7977 | 6685 |
|  | 164c | UGGAGAAGCAGGGCACGUGCG(0) | 19 | 8 |
|  | 164d | UGGAGAAGCAGGGCACGUGCU(0)r | 155 | 163 |
|  | 164e | UGGAGAAGCAGGGUAGGUGAG(1)r | 1 | 0 |
|  | 164f | UGGAGAAGCAGGGCACAUGCU | 31 | 7 |
| **165** | 165a | UCGGACCAGGCUUCAUCCCCC | 10 | 60 |
| **166** | 166a | UCGGACCAGGCUUCAUUCCCC | 3861 | 9728 |
|  | 166c | UCGGACCAGGCUUCAUUCCCCA | 22 | 44 |
|  | 166d | UCGGACCAGGCUUCAUUCCCGU | 9 | 22 |
|  | 166e | UCGAACCAGGCUUCCUUCCCC | 0 | 2 |
|  | 166g | UCGGACCAGGCUUCAUUCCUC | 29 | 76 |
|  | 166h | UCGGACCAGGCUUCAUUCC | 190 | 496 |
|  | 166j | UCUCGGACCAGGCUUCAUUCC | 799 | 1397 |
|  | 166k | UCGGACCAGGCUUCAAUCCCG | 0 | 1 |
|  | 166m | UCGGACCAGGCUUCAUUCCCU | 2 | 19 |
|  | 166n | UCGGACCAGGCUUCAUUUU | 29 | 64 |
| **167** | 167 | GGAAGCUGCCAGCAUGAUCUU | 12 | 42 |
|  | 167a | UGAAGCUGCCAGCAUGAUCUCA | 187396 | 356865 |
|  | 167c | UGAAGCUGCCAGCAUGAUCUC | 70641 | 123809 |
|  | 167d | UGAAGCUGCCAGCAUGAUCUGG | 559 | 619 |
|  | 167f | UGAAGCUGCCAGCAUGAUCUU | 35337 | 64173 |
|  | 167h | UGAAGCUGCCAACAUGAUCUC | 15 | 29 |
| **168** | 168a | UCGCUUGGUGCAGGUCGGGAA | 1633 | 1208 |
| **169** | 169a | CAGCCAAGGAUGACUUGCCGA | 194 | 256 |
|  | 169b | CAGCCAAGGAUGAUUUGCCGG | 96 | 119 |
|  | 169d | UGAGCCAAGGAUGACUUGCCGC | 2 | 1 |
|  | 169e | UAGCCAAGGAUGACUUGCCGA | 2 | 1 |
|  | 169g | UAGCCAAGAAUGACUUGCCUGC | 47 | 4 |
|  | 169h | UAGCCAAGGAUGACUUGCCUG | 112 | 28 |
|  | 169m | UAGCCAAAGAUGACUUGCCGA | 1 | 0 |
|  | 169n | UAGCCAAGAAUGACUUGCCU | 59 | 28 |
|  | 169o | GGAGCCAAGGAUGACUUGCCGC | 1 | 0 |
|  | 169r | UGAGUCGAGAAUGACUUGCCG | 2 | 0 |
|  | 169s | UCAGCCAAGGAUGACUUGCCG | 6 | 3 |
|  | 169t | CAGUCAAGGAUGACUUGCCG | 0 | 1 |
|  | 169z | CAGCCAUGAUGAUUUGCCGG | 1 | 0 |
| **170*** | 170 | UGAUUGAGCCGUGUCAAUAUC | 1 | 1 |
| **171** | 171 | UGAUUGAGUCGUGCCAAUAUC | 3 | 1 |
|  | 171a | UUGAGCCGUGCCAAUAUCACUU | 0 | 2 |
|  | 171b | UGAUUGAGCCGUGCCAAUAUC | 425 | 739 |
|  | 171c | UGACUGAGCCGUGCCAAUAUC | 0 | 3 |
|  | 171f | UUGAGCCGCGCCAAUAUCACU | 4 | 4 |
|  | 171g | UGAUUGAGCCGUGCCAAUAUUU | 1 | 1 |
| **172** | 172a | AGAAUCUUGAUGAUGCUGCAU | 2376 | 2151 |
|  | 172c | AGAAUCUUGAUGAUGCUGCAG | 18695 | 19397 |
|  | 172e | GGAAUCUUGAUGAUGCUGCAU | 6 | 2 |
|  | 172g | GGAAUCCUGAUGAUGCUGCAG | 44 | 59 |
|  | 172i | AGAAUCCUGAUGAUGCUGCAG | 62271 | 126063 |
| **319*** | 319a | UUGGACUGAAGGGAGCUCCCU | 1 | 0 |
|  | 319c | UUGGACUGAAGGGAGCUCCUU | 0 | 3 |
|  | 319e | UUGGACUGAAGGGAGCUCCU | 0 | 1 |
| **390** | 390a | AAGCUCAGGAGGGAUAGCGCC | 2150 | 2459 |
|  | 390a-3p | CGCUAUCCAUCCUGAGUUUCA | 107 | 56 |
|  | 390b | AAGCUCAGGAGGGAUAGCACC | 0 | 3 |
|  | 390c | GAGCUCAGGAGGGAUAGCGCC | 1 | 0 |
| **393** | 393a | UCCAAAGGGAUCGCAUUGAUCC | 7 | 25 |
|  | 393b | UCCAAAGGGAUCGCAUUGAUU | 59 | 110 |
|  | 393c | UCCAAAGGGAUCGCAUUGAUC | 27 | 49 |
| **394** | 394a | UUGGCAUUCUGUCCACCUCC | 232 | 313 |
| **395** | 395a | CUGAAGUGUUUGGGGGAACUC | 150 | 55 |
|  | 395p | CUGAAGCGUUUGGGGGAACUC | 0 | 1 |
| **396** | 396 | UUCCACGGCUUUCUUGAA | 6 | 3 |
|  | 396a | UUCCACAGCUUUCUUGAACUG | 8 | 7 |
|  | 396b | UUCCACAGCUUUCUUGAACUU | 139 | 175 |
|  | 396d | UCCACAGGCUUUCUUGAACUG | 0 | 1 |
|  | 396f | UUCCACGGCUUCUUGAACU | 0 | 2 |
|  | 396g | UUCCACGGCUUUCUUGAACUU | 2 | 3 |
| **397** | 397 | AUUGAGUGCAGCGUUGAUGAA | 105 | 30 |
|  | 397a | UCAUUGAGUGCAGCGUUGAUG | 32 | 12 |
| **398** | 398a | UGUGUUCUCAGGUCACCCCUU | 3 | 0 |
|  | 398b | UGUGUUCUCAGGUCGCCCCUG | 4 | 1 |
| **399** | 399a | UGCCAAAGGAGAAUUGCCCUG | 49 | 12 |
|  | 399b | UGCCAAAGGAGAGUUGCCCUG | 33 | 11 |
|  | 399d | UGCCAAUGGAGAUUUGCCCCG | 18 | 14 |
|  | 399f | UGCCAAAGGAGAUUUGCCCGG | 664 | 134 |
|  | 399g | UGCCAAUGGAGAUUUGCCCCU | 1 | 0 |
|  | 399i | CGCCAAAGGAGAAUUGCCCUG | 12 | 4 |
|  | 399j | UGCCAAAGGAGAUUUGUCCGG | 432 | 25 |
|  | 399l | CGCCAAAGGAGAGUUGCCCUU | 13 | 2 |
| **403** | 403 | UUAGAUUCACGCACAAACUCG | 13 | 21 |
| **408** | 408 | AUGCACUGCCUCUUCCCUGGC | 16 | 28 |
|  | 408b | UGCACUGCCUCUUCCCUGGCU | 0 | 3 |
| **472** | 472 | UCUUUCCUACUCCUCCCAUACC | 1 | 2 |
| **473*** | 473a | ACUCUCCCUCAAGGGCUUCCC | 10 | 4 |
| **477*** | 477 | AUCUCCCUCAAACGCUUCCAG | 0 | 1 |
| **479*** | 479 | CGUGAUAUUGGUUCGGCUCAUC | 9 | 0 |
| **482** | 482a | UCUUUCCUACUCCUCCCAUUCC | 3 | 6 |
| **530*** | 530a | UGCAUUUGCACCUGCACCUUC | 17 | 36 |
| **535*** | 535a | UGACAACGAGAGAGAGCACGU | 634 | 1115 |
| **827** | 827 | UUAGAUGACCAUCAACAAACA | 65 | 47 |
| **829** | 829.1 | AGCUCUGAUACCAAAUGAUGUGAU | 0 | 1 |
| **858*** | 858 | UUCGUUGUCUGUUCGACCUUG | 13 | 10 |
| **894*** | 894 | GUUUCACGUCGGGUUCACCA | 607 | 785 |

“Sequence” stands for the most occurring read that is homologue to the conserved miRNA.

* Candidate miRNAs newly identified in *Gossypium hirsutum* from this study.

** Some miRNAs are annotated by miRBase without a letter suffix and for such miRNAs we listed the whole numerical identifier.
