# Supplementary material for: Enrichment of a set of microRNAs during the cotton fiber development
Source: BMC Genomics. 2009 Sep 29;10:457. doi: 10.1186/1471-2164-10-457 (PMC2760587; doi:10.1186/1471-2164-10-457)
Supplement: Additional file 4 — Additional Table S4: Predicted targets for identified candidate miRNAs. miRNAs targeting the same gene and site are grouped together. The first listed miRNA has the least mismatches. "Mm" stands for the total amount of mismatches between the first mentioned miRNA and the predicted target. "Alignment" visually represents miRNA/mRNA complementary base-pairs and mismatches for the first listed miRNA, with vertical bars and spaces as Watson-Crick base-pairs and mismatches, respectively (G:U wobbles count as mismatches). [file 1471-2164-10-457-S4.DOC]

**Additional Table 4:**

**Predicted targets for identified candidate miRNAs.**

| **miRNA** | **Mm** | | **Alignment** | **Target** | | **Target ID** | |
| --- | --- | --- | --- | --- | --- | --- | --- |
| 156a, 156g, 156k, 157d | 2 | miR 3'-CACGAGUGAGAGAAGACAGU-5'  |||||| ||||||||| ||| mRNA 5'-GUGCUCUCUCUCUUCUAUCA-3' | | | Squamosa promoter binding like protein 9 | | TA24926:[990,1009] |
| 156a, 156g, 156k, 157d | 1 | miR 3'-CACGAGUGAGAGAAGACAGU-5'  |||||| ||||||||||||| mRNA 5'-GUGCUCUCUCUCUUCUGUCA-3' | | | Squamosa promoter binding like protein 11 | | TA26720:[710,729] |
| 156a, 156g, 156k, 157d | 1 | miR 3'-CACGAGUGAGAGAAGACAGU-5'  |||||| ||||||||||||| mRNA 5'-GUGCUCUCUCUCUUCUGUCA-3' | | | Squamosa promoter binding like protein 2 | | TA26721:[1221,1240] |
| 156a, 156k | 3 | miR 3'-CACGAGUGAGAGAAGACAGU-5'  |||| ||||||||||||| mRNA 5'-ACGCUCCCUCUCUUCUGUCA-3' | | | SBP transcription factor | | TA33402:[612,631] |
| 156a, 156g, 156k, 157d | 2 | miR 3'-CACGAGUGAGAGAAGACAGU-5'  ||||| ||||||||||||| mRNA 5'-AUGCUCUCUCUCUUCUGUCA-3' | | | Squamosa promoter binding like protein 3 | | TA41153:[692,711] |
| 156a, 156g, 156k, 157d | 1 | miR 3'-CACGAGUGAGAGAAGACAGU-5'  |||||| ||||||||||||| mRNA 5'-GUGCUCUCUCUCUUCUGUCA-3' | | | Squamosa promoter binding protein homologue 4 | | TA41550:[892,911] |
| 156a | 3 | miR 3'-CACGAGUGAGAGAAGACAGU-5'  |||| |||||||| ||||| mRNA 5'-GUGCGCACUCUCUCGUGUCA-3' | | | Cytokinin independent 2 | | CD809332:[23,42] |
| 157a | 2 | miR 3'-CACGAGAGAUAGAAGACAGUU-5'  ||||||||| |||||| |||| mRNA 5'-GUGCUCUCUCUCUUCUAUCAA-3' | | | Squamosa promoter binding like protein 9 | | TA24926:[990,1010] |
| 157a, 156b, 156c, 156d, 156h | 1 | miR 3'-CACGAGAGAUAGAAGACAGUU-5'  ||||||||| ||||||||||| mRNA 5'-GUGCUCUCUCUCUUCUGUCAA-3' | | | Squamosa promoter binding like protein 11 | | TA26720:[710,730] |
| 157a, 156b, 156c, 156d, 156h | 1 | miR 3'-CACGAGAGAUAGAAGACAGUU-5'  ||||||||| ||||||||||| mRNA 5'-GUGCUCUCUCUCUUCUGUCAA-3' | | | Squamosa promoter binding like protein 2 | | TA26721:[1221,1241] |
| 157a, 156b, 156c, 156d, 156h | 2 | miR 3'-CACGAGAGAUAGAAGACAGUU-5'  |||||||| ||||||||||| mRNA 5'-AUGCUCUCUCUCUUCUGUCAA-3' | | | Squamosa promoter binding like protein 3 | | TA41153:[692,712] |
| 157a, 156b, 156c, 156d, 156h | 1 | miR 3'-CACGAGAGAUAGAAGACAGUU-5'  ||||||||| ||||||||||| mRNA 5'-GUGCUCUCUCUCUUCUGUCAA-3' | | | Squamosa promoter binding protein homologue 4 | | TA41550:[892,912] |
| 157d | 3 | miR 3'-CACGAGAGAUAGAAGACAGU-5'  |||| ||||| |||||||| mRNA 5'-CUGCUUUCUAUGUUCUGUCA-3' | | | Triacylglycerol lipase 1 precursor | | TA24274:[1390,1409] |
| 157d | 3 | miR 3'-CACGAGAGAUAGAAGACAGU-5'  |||||||||||||||| |  mRNA 5'-GUGCUCUCUAUCUUCUUGCU-3' | | | Importin beta2 | | CO490924:[251,270] |
| 160a, 160f, 160h | 1 | miR 3'-ACCGUAUGUCCCUCGGUCCGU-5'  |||||| |||||||||||||| mRNA 5'-UGGCAUGCAGGGAGCCAGGCA-3' | | | Auxin response factor 10 | | TA37986:[429,449] |
| 160f | 3 | miR 3'-ACCGUAAGUCCCUCGGUCCGU-5'  || |||||| ||| ||||||| mRNA 5'-UGCCAUUCAUGGAUCCAGGCA-3' | | | 22 0 kDa class IV heat shock protein precursor | | TA31413:[128,148] |
| 164a, 164c, 164d, 164f | 3 | miR 3'-ACGUGCACGGGACGAAGAGGU-5'  ||||||| |||| ||||||| mRNA 5'-AGCACGUGUCCUGUUUCUCCA-3' | | | No apical meristem (NAM) protein | | TA38654:[925,945] |
| 164e | 3 | miR 3'-GAGUGGAUGGGACGAAGAGGU-5'  |||||||| | |||||||| | mRNA 5'-CUCACCUAUCUUGCUUCUCAA-3' | | | Protein kinase like protein | | TA33226:[970,990] |
| 164e | 3 | miR 3'-GAGUGGAUGGGACGAAGAGGU-5'  ||| |||||||| ||||| || mRNA 5'-CUCUCCUACCCUCCUUCUUCA-3' | | | Putative senescence associated protein | | DN826500:[329,349] |
| 165a | 3 | miR 3'-CCCCCUACUUCGGACCAGGCU-5'  ||||||||||||||||||  mRNA 5'-CUGGGAUGAAGCCUGGUCCGG-3' | | | Class III HD Zip protein 5 | | TA39412:[197,217] |
| 165a | 3 | miR 3'-CCCCCUACUUCGGACCAGGCU-5'  ||||||||||||||||||  mRNA 5'-CUGGGAUGAAGCCUGGUCCGG-3' | | | Class III HD Zip protein 8 | | TA43816:[167,187] |
| 166a, 166g, 166k, 166m | 3 | miR 3'-CCCCUUACUUCGGACCAGGCU-5'  ||||||||||||||||||  mRNA 5'-CUGGAAUGAAGCCUGGUCCGG-3' | | | Class III HD Zip protein 5 | | TA34377:[279,299] |
| 166g | 3 | miR 3'-CUCCUUACUUCGGACCAGGCU-5'  |||||||||| || |||||| mRNA 5'-GAGGAAUGAACCCAAGUCCGA-3' | | | ARF GTPase activating protein Medicago | | TA36739:[747,767] |
| 166h | 3 | miR 3'-CCUUACUUCGGACCAGGCU-5'  | ||||||||||||| || mRNA 5'-GCAAUGAAGCCUGGUGUGA-3' | | | X Pro dipeptidase like protein | | TA28761:[192,210] |
| 166h | 2 | miR 3'-CCUUACUUCGGACCAGGCU-5'  || |||||||||||||||  mRNA 5'-GGGAUGAAGCCUGGUCCGG-3' | | | Class III HD Zip protein 8 | | TA43816:[169,187] |
| 166h | 3 | miR 3'-CCUUACUUCGGACCAGGCU-5'  ||| |||| |||||||| | mRNA 5'-GGACUGAACCCUGGUCCAA-3' | | | nucleotide binding Arabidopsis thaliana | | DR453001:[741,759] |
| 166n | 3 | miR 3'-UUUUACUUCGGACCAGGCU-5'  | ||| |||||||||||| mRNA 5'-ACGAUGUAGCCUGGUCCGA-3' | | | Peroxisomal targeting signal 2 receptor | | TA28361:[272,290] |
| 167, 167f | 3 | miR 3'-UUCUAGUACGACCGUCGAAGG-5'  ||||||| |||||||||||  mRNA 5'-AAGAUCAGGCUGGCAGCUUGU-3' | | | Auxin response factor 8 | | TA29093:[488,508] |
| 167a | 3 | miR 3'-ACUCUAGUACGACCGUCGAAGU-5'  |||||||| |||||||||||  mRNA 5'-UGAGAUCAGGCUGGCAGCUUGU-3' | | | Auxin response factor 4 | | TA34835:[243,264] |
| 167h | 3 | miR 3'-CUCUAGUACAACCGUCGAAGU-5'  ||||| ||||| |||||| || mRNA 5'-GAGAUGAUGUUUGCAGCUGCA-3' | | | Photosystem II 5 kDa protein chloroplast | | TA26382:[258,278] |
| 168a | 3 | miR 3'-AAGGGCUGGACGUGGUUCGCU-5'  |||||| ||||||||||| | mRNA 5'-AUCCCGAGCUGCACCAAGCAA-3' | | | AGO1 1 | | TA20937:[351,371] |
| 169b | 3 | miR 3'-GGCCGUUUAGUAGGAACCGAC-5'  ||||||||||| |||||||  mRNA 5'-GCGGCAAAUCAUUCUUGGCUU-3' | | | Nuclear transcription factor Y subunit A 9 | | TA25614:[469,489] |
| 169b | 3 | miR 3'-GGCCGUUUAGUAGGAACCGAC-5'  | ||||||||||| ||||| | mRNA 5'-CGGGCAAAUCAUCUUUGGCAG-3' | | | Putative MYB family transcription factor | | TA33066:[537,557] |
| 169d, 169o | 2 | miR 3'-CGCCGUUCAGUAGGAACCGAGU-5'  | || ||||||||||||||||| mRNA 5'-GUGGGAAGUCAUCCUUGGCUCA-3' | | | Nuclear transcription factor Y subunit A 3 | | DT460601:[668,689] |
| 169h | 3 | miR 3'-GUCCGUUCAGUAGGAACCGAU-5'  | || |||||||||||||||  mRNA 5'-CUGGGAAGUCAUCCUUGGCUC-3' | | | Nuclear transcription factor Y subunit A 5 | | TA42159:[796,816] |
| 169t | 3 | miR 3'-GCCGUUCAGUAGGAACUGAC-5'  |||||||| | |||||||| mRNA 5'-CGGCAAGUUAAUCUUGACUG-3' | | | Putative cinnamyl alcohol dehydrogenase | | TA21937:[709,728] |
| 171a | 3 | miR 3'-UUCACUAUAACCGUGCCGAGUU-5'  |||||||||||| |||||| | mRNA 5'-AAGUGAUAUUGGAUCGGCUCCA-3' | | | APETAL2 SCARECROW like protein | | TA37602:[526,547] |
| 172a | 3 | miR 3'-UACGUCGUAGUAGUUCUAAGA-5'  | |||| || ||||||||||| mRNA 5'-ACGCAGGAUGAUCAAGAUUCU-3' | | | Ser Thr specific protein phosphatase 2A A regulatory subunit beta isoform | | TA25438:[672,692] |
| 172a, 172c, 172e, 172g, 172i | 3 | miR 3'-UACGUCGUAGUAGUUCUAAGA-5'  ||||||||||||| |||||  mRNA 5'-CUGCAGCAUCAUCAGGAUUCC-3' | | | APETAL2 like protein | | TA33167:[915,935] |
| 172i | 3 | miR 3'-GACGUCGUAGUAGUCCUAAGA-5'  ||||||||||| |||||| | mRNA 5'-CUGCAGCAUCAAUAGGAUUAU-3' | | | Mitochondrial processing peptidase | | TA38657:[419,439] |
| 393a | 2 | miR 3'-CCUAGUUACGCUAGGGAAACCU-5'  | | |||||||||||||||||| mRNA 5'-GAAACAAUGCGAUCCCUUUGGA-3' | | | Auxin responsive factor TIR1 like protein | | TA29137:[1048,1069] |
| 393a | 3 | miR 3'-CCUAGUUACGCUAGGGAAACCU-5'  | |||||||||||||||||| mRNA 5'-GAGACAAUGCGAUCCCUUUGGA-3' | | | TRANSPORT INHIBITOR RESPONSE 1 protein | | TA31729:[60,81] |
| 394a | 3 | miR 3'-CCUCCACCUGUCUUACGGUU-5'  |||| ||| ||||| ||||| mRNA 5'-GGAGCUGGCCAGAAAGCCAA-3' | | | Cytochrome P450 Citrus | | TA20163:[991,1010] |
| 395a, 395p | 3 | miR 3'-CUCAAGGGGGUUUGUGAAGUC-5'  ||||||| |||||| |||||  mRNA 5'-GAGUUCCUCCAAACUCUUCAU-3' | | | ATP sulfurylase precursor | | TA20253:[627,647] |
| 395a, 395p | 2 | miR 3'-CUCAAGGGGGUUUGUGAAGUC-5'  |||||||||||||||||||  mRNA 5'-AAGUUCCCCCAAACACUUCAA-3' | | | Serine hydroxymethyltransferase 6 | | TA38237:[58,78] |
| 395p | 3 | miR 3'-CUCAAGGGGGUUUGCGAAGUC-5'  ||||| |||||||||||||  mRNA 5'-GAGUUAACCCAAACGCUUCAA-3' | | | Sulfate transporter 2 1 | | TA44237:[17,37] |
| 396 | 3 | miR 3'-AAGUUCUUUCGGCACCUU-5'  |||||||||| | |||| mRNA 5'-UUCAAGAAAGUUGCGGAA-3' | | | 39 kDa calcium-binding EF hand family protein | | TA23921:[838,855] |
| 396 | 3 | miR 3'-AAGUUCUUUCGGCACCUU-5'  || ||||| ||||| ||| mRNA 5'-UUUAAGAAUGCCGUUGAA-3' | | | 2 3 bisphosphoglycerate independent phosphoglycerate mutase | | TA25824:[788,805] |
| 396 | 3 | miR 3'-AAGUUCUUUCGGCACCUU-5'  || |||| ||||| |||| mRNA 5'-UUGAAGACAGCCGCGGAA-3' | | | Putative translation initiation factor EIF 2B alpha subunit | | TA26571:[755,772] |
| 396 | 3 | miR 3'-AAGUUCUUUCGGCACCUU-5'  |||||||| |||| ||| mRNA 5'-AUCAAGAAAACCGUCGAA-3' | | | Putative membrane related protein CP5 | | TA27524:[515,532] |
| 396 | 3 | miR 3'-AAGUUCUUUCGGCACCUU-5'  ||||||||| | ||||| mRNA 5'-UUCAAGAAACACAUGGAA-3' | | | HMG I and HMG Y DNA binding | | TA31719:[226,243] |
| 396 | 3 | miR 3'-AAGUUCUUUCGGCACCUU-5'  |||| |||||| ||||| mRNA 5'-UUCACCAAAGCCAUGGAA-3' | | | Leucine Rich Repeat family protein | | TA34458:[329,346] |
| 396 | 3 | miR 3'-AAGUUCUUUCGGCACCUU-5'  | |||||||||| |||| mRNA 5'-UCCAAGAAAGCCCAGGAA-3' | | | MCM protein like protein | | TA34636:[278,295] |
| 396 | 3 | miR 3'-AAGUUCUUUCGGCACCUU-5'  || || ||||| |||||| mRNA 5'-UUUAAAAAAGCGGUGGAA-3' | | | Putative guanylate cyclase | | TA34878:[384,401] |
| 396 | 3 | miR 3'-AAGUUCUUUCGGCACCUU-5'  ||||| ||||| ||||| mRNA 5'-AUCAAGGAAGCCUUGGAA-3' | | | Gaiacol peroxidase | | TA35486:[410,427] |
| 396 | 3 | miR 3'-AAGUUCUUUCGGCACCUU-5'  |||| |||||| ||||| mRNA 5'-UUCACUAAAGCCCUGGAA-3' | | | GDSL motif lipase hydrolase like protein | | TA35819:[720,737] |
| 396 | 3 | miR 3'-AAGUUCUUUCGGCACCUU-5'  |||| ||||| |||||| mRNA 5'-GUCAAAAAAGCUGUGGAA-3' | | | Type IIB calcium ATPase | | TA40559:[246,263] |
| 396 | 3 | miR 3'-AAGUUCUUUCGGCACCUU-5'  |||||||| ||||| || mRNA 5'-UUCAAGAAUGCCGUUCAA-3' | | | Protein ABIL2 (ABL Interactor-Like) | | TA42587:[664,681] |
| 396 | 3 | miR 3'-AAGUUCUUUCGGCACCUU-5'  ||||| ||||||| ||| mRNA 5'-GUCAAGCAAGCCGUCGAA-3' | | | Helix-hairpin-helix DNA-binding motif | | AI054684:[158,175] |
| 396 | 3 | miR 3'-AAGUUCUUUCGGCACCUU-5'  |||||||| | |||||| mRNA 5'-UUCAAGAAGCCUGUGGAA-3' | | | Transcription activator | | AI055588:[519,536] |
| 396 | 3 | miR 3'-AAGUUCUUUCGGCACCUU-5'  ||||||||||| || || mRNA 5'-UUCAAGAAAGCUUUGCAA-3' | | | Peptidyl prolyl cis trans isomerase CYP40 | | AI055681:[388,405] |
| 396 | 2 | miR 3'-AAGUUCUUUCGGCACCUU-5'  ||||||||||| || ||| mRNA 5'-UUCAAGAAAGCGGUCGAA-3' | | | Neutral ceramidase precursor | | AI731269:[331,348] |
| 396 | 3 | miR 3'-AAGUUCUUUCGGCACCUU-5'  |||| ||||||||||| mRNA 5'-AUCAAACAAGCCGUGGAA-3' | | | protease inhibitor/seed storage/lipid transfer protein (LTP) family protein | | DN827878:[230,247] |
| 396 | 3 | miR 3'-AAGUUCUUUCGGCACCUU-5'  |||| ||||||||||| mRNA 5'-AUCAAACAAGCCGUGGAA-3' | | | protease inhibitor/seed storage/lipid transfer protein (LTP) family protein | | DN827885:[206,223] |
| 396 | 3 | miR 3'-AAGUUCUUUCGGCACCUU-5'  ||||||| ||| ||||| mRNA 5'-UUCAAGAGAGCAAUGGAA-3' | | | Putative MYB family transcription factor | | DR455107:[377,394] |
| 396 | 3 | miR 3'-AAGUUCUUUCGGCACCUU-5'  |||||||||| | |||| mRNA 5'-UUCAAGAAAGUUGCGGAA-3' | | | Putative EF hand containing protein | | DR456538:[450,467] |
| 396 | 3 | miR 3'-AAGUUCUUUCGGCACCUU-5'  | |||||||| |||||| mRNA 5'-CUUAAGAAAGCUGUGGAA-3' | | | TAT binding protein homolog (twin arginine translocation/membrane transporter) | | DT048786:[272,289] |
| 396 | 3 | miR 3'-AAGUUCUUUCGGCACCUU-5'  | |||||| |||||||| mRNA 5'-GUGAAGAAAACCGUGGAA-3' | | | Putative PRL1 (pleiotropic regulatory locus) associated protein | | DT468691:[388,405] |
| 396a | 3 | miR 3'-GUCAAGUUCUUUCGACACCUU-5'  ||||||||| |||||| ||| mRNA 5'-CAGUUCAAGCAAGCUGAAGAA-3' | | | Pyrophosphate dependent phosphofructokinase beta subunit | | TA21978:[606,626] |
| 396a | 3 | miR 3'-GUCAAGUUCUUUCGACACCUU-5'  ||||||||| |||||| ||| mRNA 5'-CAGUUCAAGCAAGCUGAAGAA-3' | | | Pyrophosphate fructose 6 phosphate 1 phosphotransferase subunit beta | | TA21979:[604,624] |
| 396a | 3 | miR 3'-GUCAAGUUCUUUCGACACCUU-5'  ||||||| || ||||||||| mRNA 5'-CAGUUCAUGAGGGCUGUGGAA-3' | | | Putative transcriptional regulator | | TA33147:[377,397] |
| 396d | 3 | miR 3'-GUCAAGUUCUUUCGGACACCU-5'  ||||| ||||||| || |||| mRNA 5'-CAGUUGAAGAAAGUCUUUGGA-3' | | | Glutathione transferase 8 | | TA24033:[691,711] |
| 396d | 3 | miR 3'-GUCAAGUUCUUUCGGACACCU-5'  ||||||| | |||| |||||| mRNA 5'-CAGUUCAGGCAAGCUUGUGGA-3' | | | myc-related transcription factor 1 | | TA28027:[371,391] |
| 396d | 1 | miR 3'-GUCAAGUUCUUUCGGACACCU-5'  | ||||||||||||||||||| mRNA 5'-CCGUUCAAGAAAGCCUGUGGA-3' | | | RWP-RK domain-containing protein | | TA28530:[759,779] |
| 396d | 3 | miR 3'-GUCAAGUUCUUUCGGACACCU-5'  |||||||||||| |||||| mRNA 5'-GCGUUCAAGAAAGCUUGUGGA-3' | | | Growth regulating factor 12 | | TA41245:[559,579] |
| 396d | 2 | miR 3'-GUCAAGUUCUUUCGGACACCU-5'  ||||||||||||||||||| mRNA 5'-GCGUUCAAGAAAGCCUGUGGA-3' | | | Growth regulating factor 9 | | TA41374:[639,659] |
| 396d | 2 | miR 3'-GUCAAGUUCUUUCGGACACCU-5'  | |||||||||||||||||| mRNA 5'-CGUUUCAAGAAAGCCUGUGGA-3' | | | Putative transcription activator | | TA41594:[801,821] |
| 396f | 3 | miR 3'-UCAAGUUCUUCGGCACCUU-5'  ||||| ||||| |||||| mRNA 5'-UGUUCAUGAAGCAGUGGAA-3' | | | Ribonucleotide reductase small subunit | | TA22084:[785,803] |
| 396f | 2 | miR 3'-UCAAGUUCUUCGGCACCUU-5'  |||||| ||||| |||||| mRNA 5'-AGUUCAUGAAGCAGUGGAA-3' | | | Brefeldin A sensitive Golgi protein like | | TA29159:[196,214] |
| 396f | 3 | miR 3'-UCAAGUUCUUCGGCACCUU-5'  |||||||||| |||| || mRNA 5'-UGUUCAAGAAGACGUGCAA-3' | | | Lysine and histidine specific transporter | | TA36574:[268,286] |
| 396f | 3 | miR 3'-UCAAGUUCUUCGGCACCUU-5'  ||||| ||||||| ||||  mRNA 5'-AGUUCCAGAAGCCAUGGAG-3' | | | Mg2 transporter protein CorA | | TA44305:[59,77] |
| 396f | 3 | miR 3'-UCAAGUUCUUCGGCACCUU-5'  ||| |||||||| ||||| mRNA 5'-AGUACAAGAAGCUAUGGAA-3' | | | Histone lysine N methyltransferase ATXR2 | | AI727558:[418,436] |
| 396f | 3 | miR 3'-UCAAGUUCUUCGGCACCUU-5'  || ||||||||||||||  mRNA 5'-GGUACAAGAAGCCGUGGAU-3' | | | RNA polymerase beta | | DN827614:[25,43] |
| 396f | 3 | miR 3'-UCAAGUUCUUCGGCACCUU-5'  |||||||| ||| ||||| mRNA 5'-UGUUCAAGACGCCAUGGAA-3' | | | Ac transposase like protein | | DR453659:[528,546] |
| 396f | 3 | miR 3'-UCAAGUUCUUCGGCACCUU-5'  || ||||||||| || ||| mRNA 5'-AGAUCAAGAAGCAGUUGAA-3' | | | Lecithin:cholesterol acyltransferase family protein / LACT family protein | | DT464319:[661,679] |
| 397 | 3 | miR 3'-AAGUAGUUGCGACGUGAGUUA-5'  ||||||| |||||||| ||| mRNA 5'-CUCAUCAAUGCUGCACUUAAU-3' | | | Diphenol oxidase | | TA28947:[719,739] |
| 397 | 3 | miR 3'-AAGUAGUUGCGACGUGAGUUA-5'  || ||||| |||||||| ||| mRNA 5'-UUAAUCAAUGCUGCACUUAAU-3' | | | Laccase (copper-containing oxidase enzym) | | TA31121:[729,749] |
| 398a | 3 | miR 3'-UUCCCCACUGGACUCUUGUGU-5'  |||||||| |||||| |||| mRNA 5'-AAGGGGUGCUCUGAGAUCACA-3' | | | Cytoplasmic Cu ZnSOD | | TA21436:[82,102] |
| 398a | 3 | miR 3'-UUCCCCACUGGACUCUUGUGU-5'  |||||||| |||||| |||| mRNA 5'-AAGGGGUGCUCUGAGAUCACA-3' | | | Superoxide dismutase | | CO496437:[29,49] |
| 399a, 399f | 3 | miR 3'-GUCCCGUUAAGAGGAAACCGU-5'  | ||||||| ||||||||| | mRNA 5'-CCGGGCAAUGCUCCUUUGGUA-3' | | | Clathrin heavy chain putative expressed | | TA39867:[635,655] |
| 399f | 3 | miR 3'-GGCCCGUUUAGAGGAAACCGU-5'  |||||||||| |||||||| mRNA 5'-ACGGGCAAAUCAUCUUUGGCA-3' | | | Putative MYB family transcription factor | | TA33066:[536,556] |
| 399g | 3 | miR 3'-UCCCCGUUUAGAGGUAACCGU-5'  |||||||||| | ||||| || mRNA 5'-AGGGGCAAAUAUACAUUGACA-3' | | | Vacuolar ATP synthase subunit B isoform 1 | | TA24484:[1195,1215] |
| 399g | 3 | miR 3'-UCCCCGUUUAGAGGUAACCGU-5'  ||||||||||| | |||||| mRNA 5'-AGGGGCAAAUCAUCUUUGGCA-3' | | | MYB transcription factor | | TA29368:[319,339] |
| 399g | 3 | miR 3'-UCCCCGUUUAGAGGUAACCGU-5'  |||||||||| | ||||| || mRNA 5'-AGGGGCAAAUAUACAUUGACA-3' | | | Vacuolar ATP synthase subunit B isoform 2 | | CO495556:[145,165] |
| 408 | 3 | miR 3'-CGGUCCCUUCUCCGUCACGUA-5'  |||||| ||||||||||| | mRNA 5'-GCCAGGAUAGAGGCAGUGCUU-3' | | | Blue copper protein precursor | | TA21761:[631,651] |
| 472 | 3 | miR 3'-CCAUACCCUCCUCAUCCUUUCU-5'  |||||||| ||| | ||||||| mRNA 5'-GGUAUGGGUGGACUUGGAAAGA-3' | | | Disease resistance protein RGA2 | | TA42529:[692,713] |
| 477 | 3 | miR 3'-GACCUUCGCAAACUCCCUCUA-5'  |||||||| ||||||||| | mRNA 5'-CUGGAAGCUGUUGAGGGAGGU-3' | | | Putative DNA binding protein PD3 chloroplast | | TA38189:[165,185] |
| 858 | 3 | miR 3'-GUUCCAGCUUGUCUGUUGCUU-5'  | ||| ||||||||||| ||| mRNA 5'-CCAGGGCGAACAGACAAUGAA-3' | | | MYB like DNA binding domain protein | | TA30693:[349,369] |
| 858 | 3 | miR 3'-GUUCCAGCUUGUCUGUUGCUU-5'  | ||| ||||||||||| ||| mRNA 5'-CCAGGACGAACAGACAAUGAA-3' | | | GHMYB10 | | TA35979:[337,357] |

miRNAs targeting the same gene and site are grouped together. The first listed miRNA has the least mismatches. “Mm” stands for the total amount of mismatches between the first mentioned miRNA and the predicted target. “Alignment” visually represents miRNA/mRNA complementary base-pairs and mismatches for the first listed miRNA, with vertical bars and spaces as Watson-Crick base-pairs and mismatches, respectively (G:U wobbles count as mismatches).
